# ZmmiR169q/ZmNF-YA8 is a module that homeostatically regulates primary root growth and salt tolerance in maize

**DOI:** 10.3389/fpls.2023.1163228

**Published:** 2023-06-28

**Authors:** Lijuan Xing, Lan Zhang, Hongyan Zheng, Zhuoxia Zhang, Yanzhong Luo, Yuan Liu, Lei Wang

**Affiliations:** ^1^Biotechnology Research Institute, Chinese Academy of Agricultural Sciences (CAAS), Beijing, China; ^2^National Nanfan Research Institute (Sanya), Chinese Academy of Agricultural Sciences (CAAS), Hainan, China

**Keywords:** maize (*Zea mays*. L), salt tolerance, mir169, NF-YA, auxin

## Abstract

In response to salt stress, plants alter the expression of manifold gene networks, enabling them to survive and thrive in the face of adversity. As a result, the growth and development of plant roots could be drastically altered, with significant inhibition of the growth of root meristematic zones. Although it is known that root growth is primarily regulated by auxins and cytokinins, the molecular regulatory mechanism by which salt stress stunts root meristems remains obscure. In this study, we found that the ZmmiR169q/ZmNF-YA8 module regulates the growth of maize taproots in response to salt stress. Salt stress downregulates ZmmiR169q expression, allowing for significant upregulation of ZmNF-YA8, which, in turn, activates ZmERF1B, triggering the upregulation of ASA1 and ASA2, two rate-limiting enzymes in the biosynthesis of tryptophan (Trp), leading to the accumulation of auxin in the root tip, thereby inhibiting root growth. The development of the maize root is stymied as meristem cell division and meristematic zone expansion are both stifled. This study reveals the ZmmiR169q/ZmNF-YA8 module’s involvement in maintaining an equilibrium in bestowing plant salt tolerance and root growth and development under salt stress, providing new insights into the molecular mechanism underlying the homeostatic regulation of plant development in response to salt stress.

## 1 Introduction

Roots are the primary plant organs that sense salinity; as a result, salt stress directly impacts root growth and development (Wang et al., 2009a). The maize root system encompasses several root types, including the embryonic primary roots, seminal roots (Wang et al., 2023), and postembryonic crown and brace roots that originate from below-ground nodes (Hochholdinger et al., 2004). Primary roots form during embryonic development and expand shortly after seed germination. As crucial components of the root system, primary roots uptake water and mineral nutrients while providing mechanical support for plant development (Zheng et al., 2016; Qin et al., 2017). In plants, the root system initiates with the emergence of primary roots during zygotic embryogenesis (Macgregor et al., 2008). The root meristem plays an imperative role in maintaining root growth, as meristematic cells at the root apex regulate root development and the formation of all underground organs (Perilli et al., 2012). Further, root development is influenced by phytohormones and various environmental factors (Sun et al., 2017).

High soil salinity has devastating consequences for agricultural crops, stunting their growth and development and ultimately leading to yield loss and crop failure (Zhang et al., 2016). The most noticeable effect of salt stress is the inhibition of root development (Yuan et al., 2016). Typically, excess salt in the soil inhibits root elongation by hindering cell expansion in the root elongation zone (West et al., 2004b; Bustos et al., 2008) and impeding root meristematic activity (West et al., 2004a). It has been documented that salt stress results in a decrease in the size of the root meristem by increasing nitric oxide (NO) accumulation, which inhibits *PINFORMED* (*PIN*) expression and stabilizes the expression of *IAA17*, thereby reducing auxin levels and inhibiting auxin signal transduction (Liu et al., 2015b).

Auxin and ethylene are phytohormones that play an important role in plant root growth and development (Strader et al., 2009). Ethylene impacts root growth by regulating auxin biosynthesis and transport-dependent auxin distribution (Ruzicka et al., 2007). There are a number of active natural auxins in plants, among which IAA is perhaps the most extensively studied (Korasick et al., 2013). IAA biosynthesis can be dichotomized into two primary pathways, one of which is Trp-independent and the other of which is Trp-dependent (Woodward and Bartel, 2005; Mano and Nemoto, 2012; Zhao, 2012). *WEAK ETHYLENE INSENSITIVE2/ANTHRANILATE SYNTHASE α1* (*WEI2/ASA1*)*, WEAK ETHYLENE INSENSITIVE7/ANTHRANILATE SYNTHASE β 1* (*WEI7/ASB1*)*, TRYPTOPHAN AMINOTRANSFERASE OF ARABIDOPSIS* (*TAA1*), and TRYPTOPHAN AMINOTRANSFERASE RELATED1 (*TAR1*) are a few auxin biosynthetic genes, whose transcription in roots is activated by ethylene treatment (Stepanova et al., 2005; Stepanova et al., 2008). *WEI2* and *WEI7* encode the α and β subunits of anthranilate synthase, respectively, a rate-limiting enzyme in the biosynthesis of the auxin precursor Trp (Stepanova et al., 2008). As a downstream transcription factor in the ethylene signaling pathway, ERF1 transcription is activated through EIN3/EIL binding to a specific sequence within its promoter (Ren et al., 2017), and in turn, it directly upregulates ASA1, boosting auxin accumulation and ethylene-induced suppression of root development (Mao et al., 2016). Such a regulatory mechanism by which ethylene augments auxin biosynthesis and modulates root growth is crucial to plant development in the face of environmental adversaries, including but not limited to salt stress (Cao et al., 2007; Cheng et al., 2013).

The nuclear factor-Y (NF-Y) complex, a group of transcriptional regulators conserved among all eukaryotic organisms, plays an active role in regulating a bewildering array of plant growth and development processes (Mantovani, 1999). Specifically, NF-YA interacts with the cis-element CCAAT box in the promoter of downstream genes in a sequence-specific manner (Xing et al., 1993). In Arabidopsis, NF-YA2 and NF-YA10 modulate leaf development by regulating the YUC2-mediated auxin signaling pathway (Zhang et al., 2017). NF-YA is known to be the primary target of miR169 in plants (Rhoades et al., 2002). In maize, Zma-miR169o inhibits the expression of *ZmNF-YA13* and activates the expression of *ZmYUC1*, leading to increases in grain size and grain weight as the result of an increase in the number of endosperm cells (Zhang et al., 2022a). Likewise, regulated by miR169defg isoforms, Arabidopsis NF-YA2 and NF-YA10 modulate the length of the root apical meristem (RAM) as well as the initiation and density of the lateral roots (LR) (Sorin et al., 2014). However, the regulatory role of NF-YA in root development has so far been documented only in Arabidopsis. Given the tremendous complexity of the NF-YA regulatory network, which may vary between species, it is imperative to evaluate its effects on individual species rather than assuming identical behavior across all. Therefore, exploring the molecular processes underpining NF-YA’s role in maize root growth in response to salt stress is clearly warranted.

Our previous research has demonstrated that maize ZmmiR169q targets *ZmNF-YA8*, and the attenuation of maize miR169q expression due to salt stress activates ZmNF-YA8 expression (Xing et al., 2022). Consequently, primary root growth is inhibited, mirroring the phenotype of primary root growth under salt stress. However, additional research is required to thoroughly assess its functional involvement in root formation. In this study, we sought to explore the mechanism by which the ZmmiR169q/ZmNF-YA8 module regulates primary root development in maize. By analyzing transgenic maize lines overexpressing ZmNF-YA8 (ZmNF-YA8-OE), we found that the length of primary roots was closely associated with the expression level of *ZmERF1B*. ZmNF-YA8 specifically binds to a CCAAT box in the promoter of *ZmERF1B* and positively regulates its expression, which in turn expedites growth hormone biosynthesis in root tips, resulting in a dwindling of primary root growth and a shortening of root length. These findings may provide further insights into the interaction between ethylene and growth hormones in controlling root growth in maize, as well as the potential functional involvement of the ZmmiR169q/ZmNF-YA8 in this process. By employing such a mechanism, plants can maintain a judicious equilibrium between root development and salt stress tolerance.

## 2 Materials and methods

### 2.1 Plant materials

The transgenic maize lines overexpressing *ZmNF-YA8* and ZmmiR169q, termed *ZmNF-YA8*-OE and ZmmiR169q-OE, respectively, were generated as previously reported (Xing et al., 2022).

### 2.2 Plant growth and salt treatments

For plant establishment, seeds of *ZmNF-YA8*-OE, ZmmiR169q-OE, and the untransformed control were surface-sterilized by immersing in a 5% (w/v) NaClO solution for 30 min, prior to rinsing five times with sterile water. These seeds were then placed between two layers of moist brown germination papers (Cat No. SD3836S) (Anchor Paper Company, St. Paul, MN, USA), which were then rolled up and placed vertically in a 2-liter beaker with shallow water, and maintained in a controlled chamber at 28/25°C (day/night), ∼70% relative humidity, and a 16/8 h (light/dark) photoperiod, The culture solution was replaced every day until the seeds germinated. In order to assess the effect of salt treatment on the phenotype of B104 plants, germinated seeds were irrigated with a 0, 50, 100, 200 mM NaCl solution for 5 days (d) and then scored for salt tolerance. To evaluate plant phenotypic performance in response to salt treatment, germinated seeds were irrigated with a 125 mM NaCl solution and incubated for 5 d before being scored for salt tolerance. For material propagation and crossing, maize plants were cultivated at the Experimental Station of the Chinese Academy of Agricultural Sciences in Langfang, Hebei Province, and Sanya, Hainan Province, China.

### 2.3 Yucasin treatments and analysis of root growth

Plant treatment by yucasin (5-(4-chlorophenyl)-4H-1,2,4-triazole-3-thiol), which is a potent inhibitor of plant auxin biosynthesis, was performed as previously described (Qin et al., 2017). Briefly, yucasin (Aladdin, C169108, Shanghai, China) was first completely dissolved in a small volume of DMSO (Cat No.V900090-500 mL, Sigma-Aldrich, St Louis, MO, USA) before being diluted in water to 0.1 M. Maize roots for the yucasin sensitivity assay of plants were grown based on the paper roll culture method described above. In brief, maize seedlings of 3-d-old in sterile water were transferred to an aqueous solution containing 10 μM yucasin and 125 mM NaCl for 5 days. Controls were subjected to treatments containing equal amounts of DMSO and NaCl for 5 days. For phenotypic evaluation, seedlings were scanned, and the root length was measured from digitized images using ImageJ (Schindelin et al., 2012).

### 2.4 Real-time quantitative reverse transcriptase PCR

Total RNA was extracted from an approximately 0.2 g root sample using an RNA Easy Fast Plant Tissue kit (Cat No. DP452, Tiangen, Beijing, China) following the manufacturer’s instructions. Approximately 2 μg of total RNA from each sample was reverse transcribed to synthesize first-strand cDNA with HiScript II Q RT SuperMix for qPCR (GeneBio Systems, Burlington, ON, Canada), following the manufacturer’s instructions. Real-time quantitative reverse transcriptase PCR (RT-qPCR) was performed using the ABI 7500 Real Time PCR Systems (Applied Biosystems, Bedford, MA, USA) and the SYBR PrimeScript™ RT-PCR kit (Cat No. RR086A, Takara, Tokyo, Japan). The maize *Actin1* (GRMZM2G126010) gene was used as the reference gene to normalize gene expression, and the fold changes of gene expression were calculated using the 2^-ΔΔCt^ method (Livak and Schmittgen, 2001; Schindelin et al., 2012). All the RT-qPCR reactions were conducted with at least three independent biological replications. The primers used for RT-qPCR are listed in **Table S1**.

### 2.5 Observation of maize primary roots

Maize roots for the observation of plants were grown based on the paper roll culture method described above. In order to evaluate the effect of salt treatment on the phenotype of B104 plants, the germinated seeds were irrigated with 0, 50, and 200 mM NaCl solution for 5 d, respectively, and then scored for salt tolerance. For ZmNF-YA8 OE lines, they were grown in a nutrient solution without NaCl for 5 days. Take the primary roots, about 2 cm in length, derived from maize seedlings, were soaked overnight in 2 mL of 7% NaOH in a 2-mL centrifuge tube at 37°C. Following extensive rinsing with distilled water, the roots with clearly visible veins and cellular structure were placed in a solution containing 5% ethanol and 25% glycerol. The morphology of root tip cells was examined and recorded with an Invitrogen EVOS™ XL Core Imaging System (Cat No.Synergy H1, Thermo Fisher Scientific, Waltham, MA, USA).

### 2.6 *In situ* hybridization

*In situ* hybridization and immunological detection were performed as described by Zhang et al. (Zhang et al., 2022b). In brief, a 120-bp fragment, corresponding to the coding region of ZmNF-YA8, was PCR amplified and cloned into the Promega pGEM-T vector (Cat No. A1360, Promega, Madison, WI, USA). Following sequence verification, PGEM-T-ZmNF-YA8 was linearized with *Sph*I and *Sal*I, which was used as the DNA template for *in vitro* transcription using a DIG Oligonucleotide Tailing Kit (Cat No. 3353583910, Roche Diagnostics, Basel, Switzerland), which contains T7 and SP6 RNA polymerases and digoxin (DIG)-labeled UTP, generating DIG-labeled sense and antisense RNA probes (**Table S1**). These probes were used for *in situ* hybridization, as previously described (Xu et al., 2005). Slides were mounted and viewed using an Invitrogen EVOS^®^ FL Auto Imaging system (Thermo Fisher Scientific) after being exposed for 12-15 h.

### 2.7 Cytological observation

The root tips of maize primary roots were collected following salt treatment for 4 d and fixed in a 50% formalin-acetic acid-alcohol (FAA) fixative solution, before being embedded in paraffin as previously described (Qin et al., 2017). The paraffin sections of 10 µm thickness were prepared using a Leica RM2265 microtome (Leica Biosystems, Nussloch, Germany) and placed on glass slides. After staining with toluidine blue, the sections were observed and imaged using a Leica M165FC stereo microscope (Leica Biosystems, Nussloch GmbH, Germany).

### 2.8 IAA content measurement

Following treatment with 125 mM NaCl for 4 d, IAA concentration in the primary roots of B104, NF-YA8OE, and miR169q OE was determined as previously described (Luo et al., 2022). Briefly, root tips of 0.5 cm in length were collected and immediately frozen in liquid nitrogen before being pulverized into a fine powder and extracted with methanol/water/formic acid (15/4/1, v/v/v). The extracts were then evaporated to dry under nitrogen flow and reconstituted in 80% methanol (v/v). After filtration with 0.22 μm Anpel filters (PTFE, Merck Millipore, Germany), IAA concentration was analyzed using an ABSciexQTRAP6500 liquid chromatography tandem mass spectrometry (LC-MS/MS) system (Sciex, Framingham, MA, USA). All the acquired data were analyzed using MetWare (http://www.MetWare.cn).

### 2.9 RNA sequencing and transcriptome analysis

RNA was extracted from root samples derived from 2-leaf seedlings of ZmNF-YA8-OE, wild type (WT) B104, and 104-S (salt-treated WT). First-stand cDNA synthesis by reverse transcription, cDNA library construction, and subsequent RNA sequencing (RNA-seq) were performed as previously described (Xing et al., 2022). Clean data were generated by removing the reads with multiple Ns and other low-quality reads using HISAT2 (Kim et al., 2015) and mapped to the maize ‘B73’ RefGen V4 genome (http://archive.maizesequence.org/index.html). Gene expression level was estimated using the number of fragments per 1,000 base transcripts per million mapped reads (FPKM), which were calculated using CUFFLINKS (Ghosh and Chan, 2016). P < 0.05 and multiple changes > 2 (or < 0.5) were set as the thresholds for significant differential expression. The differentially expressed genes (DEGs) were analyzed by hierarchical cluster analysis to explore the transcript expression pattern. The functional classification of the genes was analyzed by enrichment of gene ontology (GO) using AGRIGov.2.0 (http://systemsbiology.cau.edu.cn/agriGOv2) with default parameters.

### 2.10 Transactivation assay in tobacco leaves

The 886-bp sequence upstream the translation initiation site (TIS, the first ATG codon) of *ZmERF1B* was amplified and inserted into pCambia1302-LUC by homologous recombination to generate the ZmERF1B:LUC reporter construct, which was subsequently introduced to *Agrobacterium tumefaciens* strain GV3101. The CDS of *ZmNF-YA8* was amplified and inserted into CaMV35S:MCS-GFP by homologous recombination to generate the 35S:ZmNF-YA8-GFP reporter construct, which was subsequently introduced to *A.* strain GV3101. The primers used for vector construction are listed in **Table S1**. *A. tumeficiens* cells harboring ZmERF1B:LUC, ZmNF-YA8-GFP, pCambia1302-LUC, and CaMV35S:MCS-GFP were suspended in 10 mM MES, 0.2 mM acetosyringone, and 10 mM MgCl_2_ in Luria Bertani (LB) medium at a density of OD_600 = _1.0, which were used to infiltrate the leaves of 5-week old *Nicotiana benthamiana* plants with a needle-free syringe. After infiltration, the plants are first maintained under low light for 1 d, followed by 2 d under high light. The infiltrated leaves were then collected, placed in a petri dish with the abaxial side up and sprayed with 1 mmol of D-fluorescein (Cat No. F3177, Promega) and 0.02% Triton X-100 aqueous solution. After dark treatment for 10 min, the leaf samples were examined and imaged using a fluorescent microscope system (Night SHADE, LB985, LB941; Berthold Technologies GmbH, Bad Wildbad, Germany).

### 2.11 Dual-luciferase assay

ZmERF1B promoter segments (1.5 kb) containing CCAAT boxes P1, P2, and P3 - in lengths of 68 bp (containing the first CCAAT box P1), 134 bp (containing the second CCAAT box P2), 650 bp (containing the third CCAAT box P3) - along with the original full length promoter fragment P0, were each cloned into the pGreenII 0800:Luc vector, generating pGreenII 0800-P0::Luc, pGreenII 0800-P1::Luc-P1, pGreenII 0800-P2::Luc, and pGreenII 0800-P3::Luc. The primers used for vector construction are listed in **Table S1**. *A. tumefaciens* GV3101 cells harboring these vectors were co-transformed into maize protoplasts with the vector pGreenII 62-SK-ZmNF-YA8, which contains the full-length coding region (CDS) of *ZmNF-YA8*. The protoplast preparation and transformation were carried out as previously described (Gao et al., 2019). After 12-16 h culture under dark conditions, the protoplasts were harvested by centrifugation at 100x g for 2 min before being subjected to a dual-luciferase assay using the Dual Luciferase Reporter Assay kit (Vazyme, Nanjing, Jiangsu Province, China) following the manufacturer’s instructions. The relative LUC activity was calculated by normalizing LUC activity to Renilla (REN) luciferase activity.

### 2.12 Electrophoretic mobility shift assay

The entire CDS of ZmNF-YA8 was cloned into the pET28a vector, generating pET28a-NF-YA8 that expresses the His-NF-YA8 fusion protein, which was then transformed into *Escherichia coli* BL21 cells. Protein expression in *E. coli* was then induced with 0.2 mM IPTG for 16 h at 16°C. Electrophoretic mobility shift assay (EMSA) was performed as previously described (Hill et al., 2019). Briefly, a DNA fragment was labeled with 5’-FAM Fluorescence in Sangon Bioteth (Shanghai, China). The probe primers used are provided in **Table S1**. The EMSA assay was performed using the Light Shift Chemiluminescent EMSA Kit (Cat No. 20148, Thermo Fisher Scientific) following the manufacturer’s instructions. Following a period of incubation for 20 min, the binding complexes were fractionated on a nondenaturing 4% polyacrylamide gel with 0.5 × TBE buffer. The gel was scanned with Tanon™ 5200Multi Chemiluminescent Imaging System (Tanon, Shanghai, China).

## 3 Results

### 3.1 Overexpressing ZmNF-YA8 inhibits the growth of primary roots in maize

**Figure 1:**
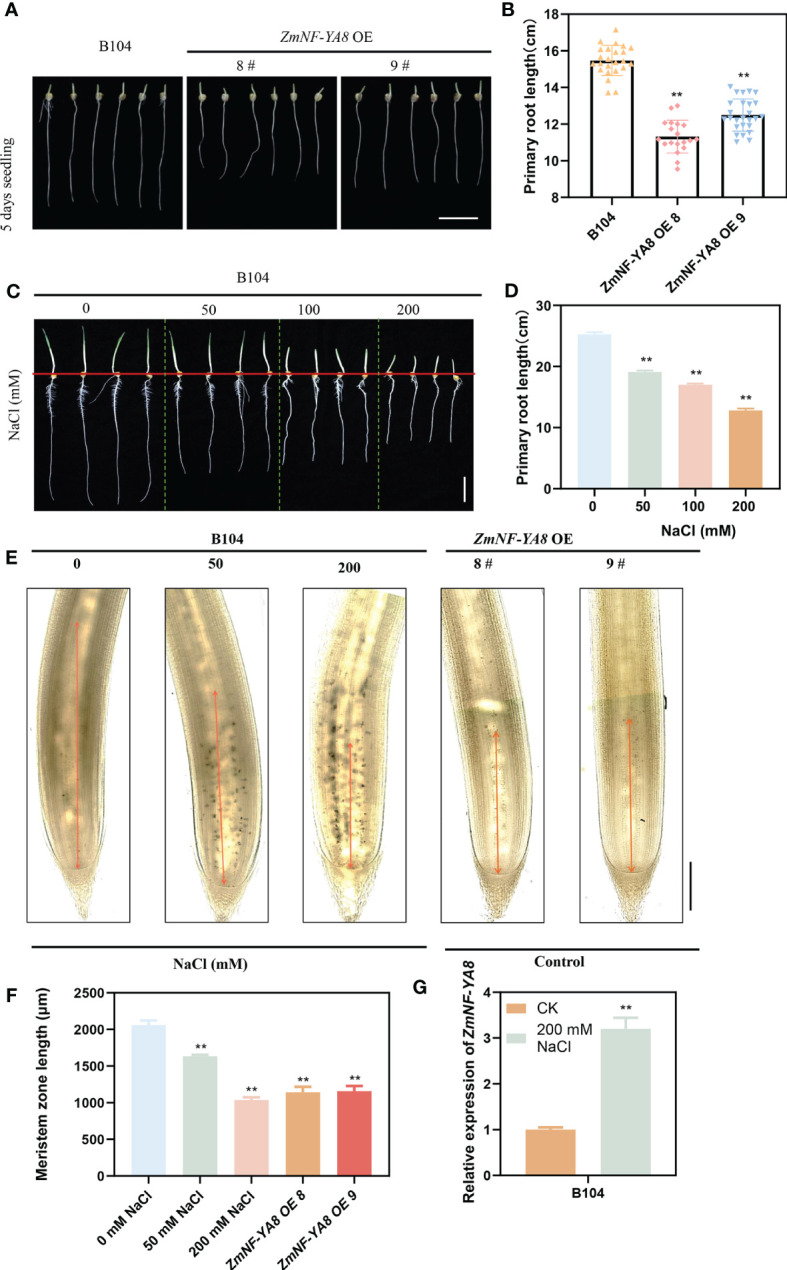
ZmNF-YA8 overexpression inhibits the growth of maize primary roots, resembling the root phenotype under salt stresses. **(A)** primary root phenotype of B104 and ZmNF-YA8-OE. Bar = 5 cm. **(B)** Root length of the plants shown in **(A)**. Values are shown as the mean ± SD of 15-20 seedlings. **(C)** primary root phenotype of B104 under different NaCl treatments. Bar = 5 cm. **(D)** Root length of the plants shown in **(C)**. Values are shown as the mean ± SD of 15-20 seedlings. **(E, F)** Phenotype and length of B104 (gradient salt treatment) and ZmNF-YA8-OE primary root meristem. Values are shown as the mean ± SD of 15-20 seedlings. **(G)** Relative expression levels of *ZmNF-YA8* in B104 under 125 mM NaCl treatment. Actin1 was used as the reference gene for quantitative PCR. Values are expressed as means ± SD of three biological repeats. Asterisks indicate significant differences (**, P < 0.01; unpaired Student’s two-tailed t-test).

As previously reported, transgenic maize overexpressing *ZmNF-YA8* (*ZmNF-YA8-*OE) displayed significant mitigation of salt stress compared to untransformed WT. Further, as is evident in **Figures 1A, B, F**, the growth of the primary roots of *ZmNF-YA8-*OE was substantially inhibited, implying the potential involvement of ZmNF-YA8 in the growth and development of maize taproots. This is well in line with previous studies showing that salt stress can inhibit root meristem activity, root cell cycle, and cell elongation (Burssens et al., 2000; West et al., 2004a), leading to retardation of primary root growth (Liu et al., 2015a). For a more nuanced understanding of the effect of salt stress on growth of maize taproot, WT B104 seedlings were exposed to various concentrations of NaCl. As shown in **Figures 1C, D**, 50, 100, and 200 mM NaCl treatments significantly inhibited the growth of the taproot, in a dosage-dependent manner. Examination of the root tip tissue of the tap root following salt treatment revealed that the length of the tap root meristematic zone decreased significantly as the salt concentration increased (**Figures 1E, F**). It appears that high concentration (100 mM) of NaCl treatment inhibited the growth of the maize primary root meristematic zone, resulting in shortened primary roots, akin to the effect of *ZmNF-YA8* overexpression under normal growth conditions.

As is evident in **Figure 1G**, the expression of *ZmNF-YA8* in the primary roots of WT B104 was significantly upregulated in response to salt treatment. This is congruent with prior studies showing that the expression of *ZmNF-YA8* was substantially enhanced as a result of salt treatment, concurrent with a significant elevation in reactive oxygen species (ROS) accumulation, which in turn attenuated the expression of ZmmiR169q (Xing et al., 2022). It is therefore conceivable that the effect of salt stress on root growth and development in maize may be governed by a ZmmiR169q/ZmNF-YA8 module.

### 3.2 ZmmiR169q/ZmNF-YA8 module regulates primary root growth in maize under salt stress

**Figure 2:**
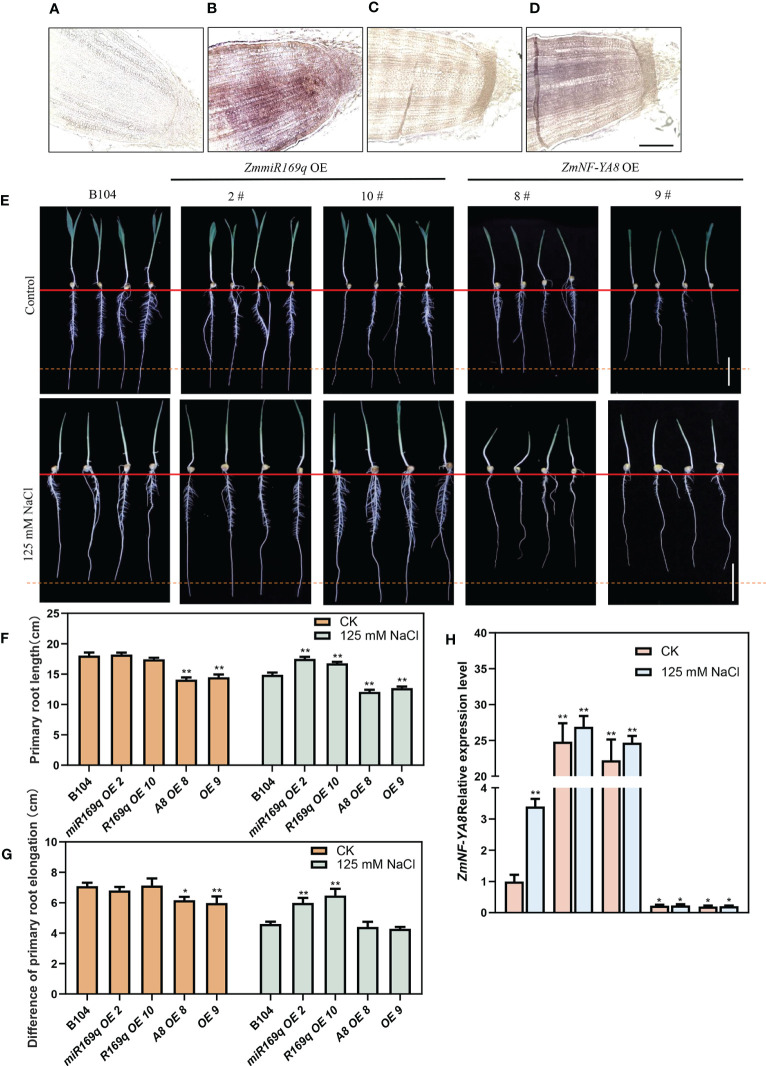
ZmmiR169q/ZmNF-YA8 module regulates primary root growth in maize under salt stress. **(A, C)** Negative control for zmmiR169q, ZmNF-YA8. Bar = 200 μm. **(B, D)** *In situ* hybridization of zmmiR169q, ZmNF-YA8, with 5 days old primary roots of WT B104. **(E)** Primary roots phenotypes of B104, ZmmiR169q-E and ZmNF-YA8-OE transgenic lines under normal and 125 mm NaCl treatment conditions. Bar = 5 cm. **(F)** Root length of the plants shown in **(E)**. **(G)** Difference primary root length (Before and after treatment of each transgenic line) in **(E)**. **(H)** Relative expression levels of ZmNF-YA8 in B104, ZmmiR169q-OE and ZmNF-YA8-OE transgenic line under normal and 125 mm NaCl treatment conditions. Actin1 was used as the reference gene for quantitative PCR. Values are expressed as means ± SD of three biological repeats. Asterisks indicate significant differences (*, P < 0.05; **, P < 0.01; unpaired Student’s two-tailed t-test).

RNA *in situ* hybridization co-localized the expressions of zma-miR169q and ZmNF-YA8 in the root apical meristem (**Figures 2A–D**). The question of whether the ZmmiR169q/ZmNF-YA8 module responds to salt stress was addressed by comparing the root phenotypes of seedlings of ZmmiR169q-OE, ZmNF-YA8-OE, and WT control B104 plants in the medium supplemented with 125 mM NaCl. As shown in **Figure 2E**, under normal growth conditions, the length of the primary roots of ZmmiR169q-OE2 and ZmmiR169q-OE10 plants was comparable to that of the control B104, but ZmNF-YA8-OE8 and ZmNF-YA8-OE9 were significantly shorter than that of B104. In contrast, under 125 mM NaCl treatment, the tap root length of ZmmiR169q-OE2 and ZmmiR169q-OE10 plants was significantly longer than that of control B104, while the tap root length of ZmNF-YA8-OE8 and ZmNF-YA8-OE9 was significantly shorter than that of B104 (**Figure 2F**). Evidently, the inhibitory effect of NaCl treatment on the growth of the primary roots was significantly less in ZmmiR169q-OE2 and ZmmiR169q-OE10 than in B104. As a result, the relative rate of root growth in ZmmiR169q-OE was much higher than in B104 (**Figure 2G**).

As shown in **Figure 2H**, without salt treatment, the expression level of ZmNF-YA8 in the primary roots of control B104 was very low. Its overexpression in ZmNF-YA8-OE plants remained significantly greater than that in ZmmiR169q-OE. In the presence of 125 mM NaCl, the expression level of ZmNF-YA8 in B104 increased by 3-fold, but it only went up 0.1 fold in the ZmNF-YA8-OE plants. It did not change at all in the ZmmiR169q OE, which shows that the overexpression of *ZmmiR169q* did not lead to the upregulation of endogenous *ZmNF-YA8* during salt stress. It is therefore tempting to assume that the ZmmiR169q/ZmNF-YA8 module is involved in the regulation of the growth and development of maize taproot in response to salt stress treatment.

### 3.3 ZmmiR169q/*ZmNF-YA8* module regulates the development of primary root meristem in maize

**Figure 3:**
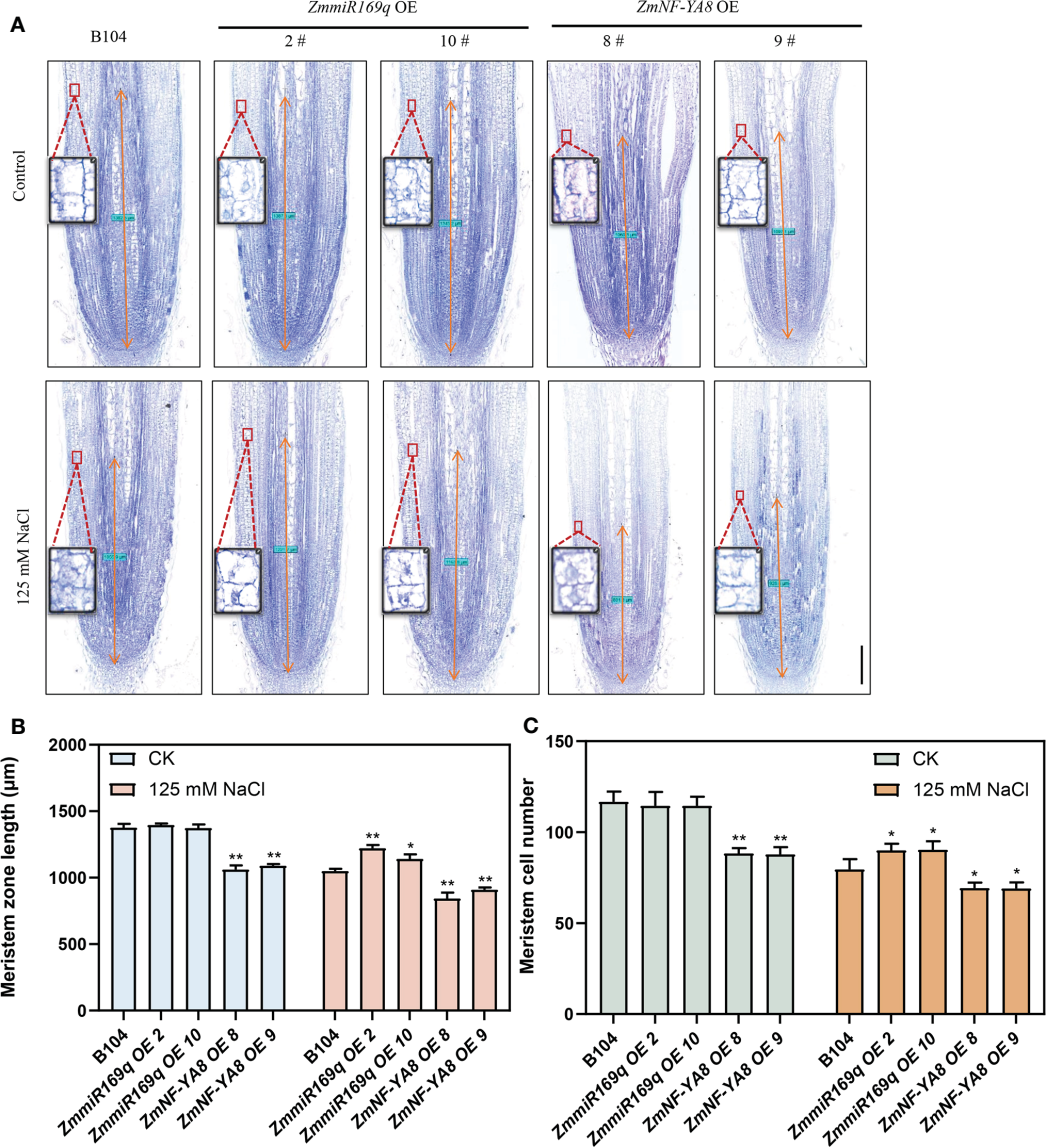
ZmmiR169q/ZmNF-YA8 module regulates the length of maize primary root meristem **(A)** Phenotype of primary root meristem in B104, ZmmiR169q-OE and ZmNF-YA8-OE transgenic line under normal and 125 mm NaCl treatment conditions Bar = 200 μm. **(B)** Meristem length of primary root in the plants shown in **(A)**. Values are shown as the mean ± SD of 10 seedlings. **(C)** Meristem cell number of primary root in the plants shown in **(A)**. Values are shown as the mean ± SD of 10 seedlings. Asterisks indicate significant differences (*, P < 0.05 **; P < 0.01; unpaired Student’s two-tailed t-test).

The homeostatic regulation of root tip cell division and differentiation of its progeny cells governs root growth (Kumar Meena et al., 2019). Research has indicated that the stress response of plant roots to salt stress is related to the suppression of the cell cycle in the meristematic zone during the onset of salt stress, resulting in shorter plant roots (Liu et al., 2015a). Additionally, salt stress can affect cell elongation, exacerbating root stunting (Burssens et al., 2000; West et al., 2004a). The root tip meristem length of ZmNF-YA8-OE8 and ZmNF-YA8-OE9 was significantly shorter than that of B104, likely due to a decrease in the number of apical meristem cells. In contrast, the root tip meristem length and cell number in the miR169q-OE plants were comparable to those in B104 (**Figure 3**). Under the condition of 125 mM NaCl treatment, the root apical meristem length and meristem cell number of ZmmiR169q-OE2 and ZmmiR169q-OE10 lines were significantly greater than those of B104, whereas those of ZmNF-YA8-OE8 and ZmNF-YA8-OE9 plants were significantly lower than B104 (**Figures 3B, C**). In light of these findings, it becomes evident that ZmmiR169q/ZmNF-YA8 regulates maize primary roots by modulating cell division in the root meristem.

### 3.4 ZmmiR169q/*ZmNF-YA8* module regulates the content of root tip auxin

**Figure 4:**
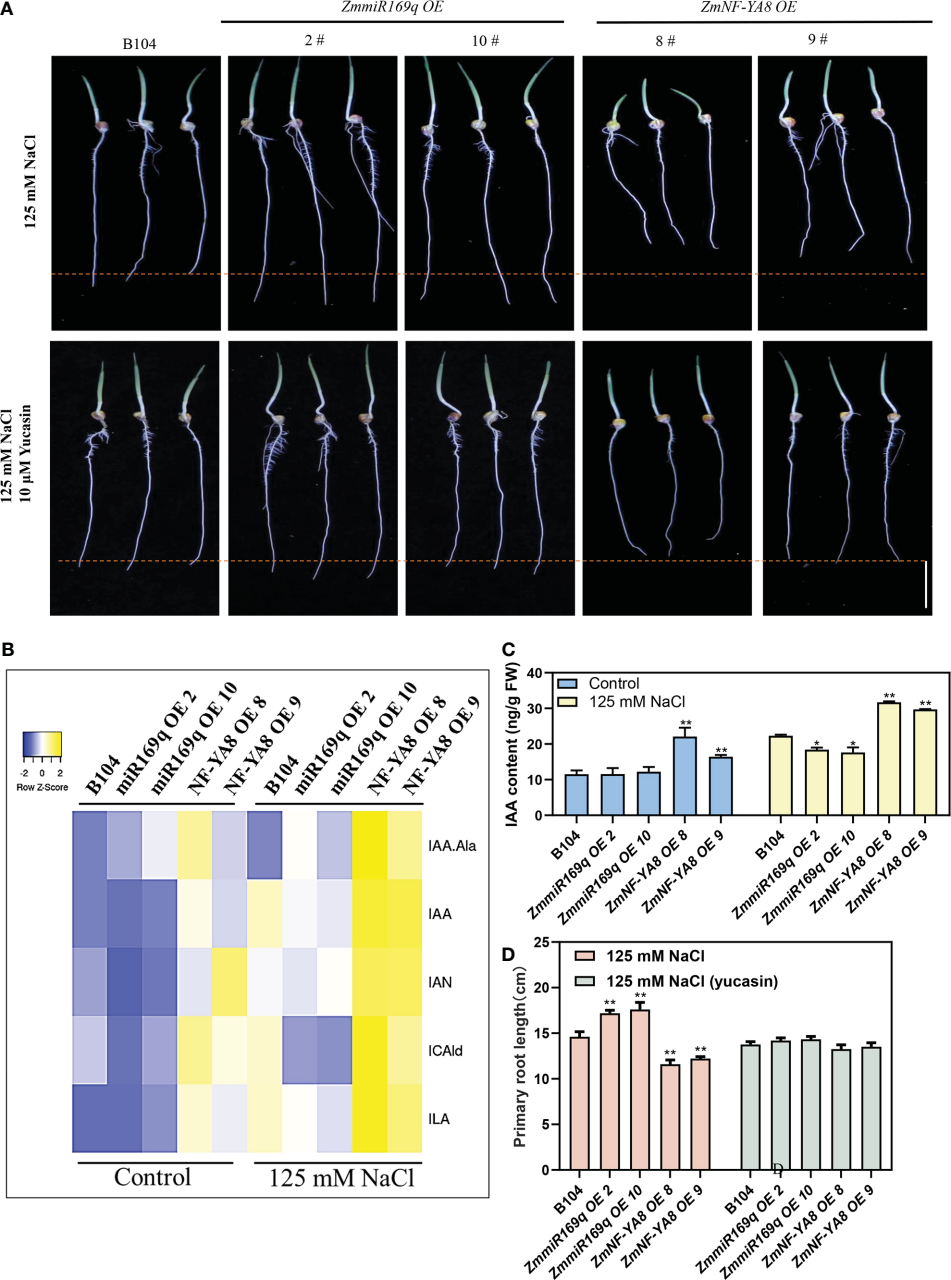
ZmmiR169q/ZmNF-YA8 module influenced the auxin content of root tip **(A)** Phenotype of primary root meristem in B104, *ZmmiR169q*-OE and *ZmNF-YA8*-OE transgenic line under 125 mM NaCl treatment and 125 mM NaCl with 10 μM yucasin. Bar = 5 cm. **(B, C)** auxin concentrations in 0.5 cm primary root from B104, *ZmmiR169q*-OE and *ZmNF-YA8*-OE transgenic line. Values are expressed as means ± SD of three biological repeats. Asterisks indicate significant differences (*, P < 0.05; **, P < 0.01; unpaired Student’s two-tailed t-test). **(D)** primary root length of the plants shown in **(A)**. Values are shown as the mean ± SD of 15-20 seedlings. Asterisks indicate significant differences (*, P < 0.05; **, P < 0.01; unpaired Student’s two-tailed t-test).

Given that phytohormones are key regulators of plant root growth and development, we analyzed the key phytohormones in the root tip cells of 1 - 2 cm zone. As shown in **Figures 4B, C**, under normal growth conditions, indole-3-acetic acid (IAA), several IAA precursors (indole-3-carboxaldehyde, 3-IAld), indole-3-acetonitrile, indole-3-acetic acid, and the IAA conjugate IAA-Ala were all significantly increased in the root tips of the ZmNF-YA8-OE8 and ZmNF-YA8-OE9 lines, but not in those of the miR169q-OE2 and miR169q-OE10 lines compared to B104. Likewise, in the presence of 125 mM NaCl, even more of these compounds were detected in the root tips of ZmNF-YA8-OE8 and ZmNF-YA8-OE9 lines, but they were significantly reduced in the root tips of miR169q-OE2 and miR169q-OE10 plants, compared to B104.

Yucasin treatment restored the taproot length in ZmNF-YA8-OE plants but inhibited the taproot growth of ZmmiR169q-OE plants and had no effect on the taproot growth in B104 (**Figures 4A, D**). This lends additional credence to the notion that the alterations in root tip growth in the ZmNF-YA8-OE plants are mediated by auxin accumulation.

### 3.5 ZmNF-YA8 activates transcription of ZmERF1B

**Figure 5:**
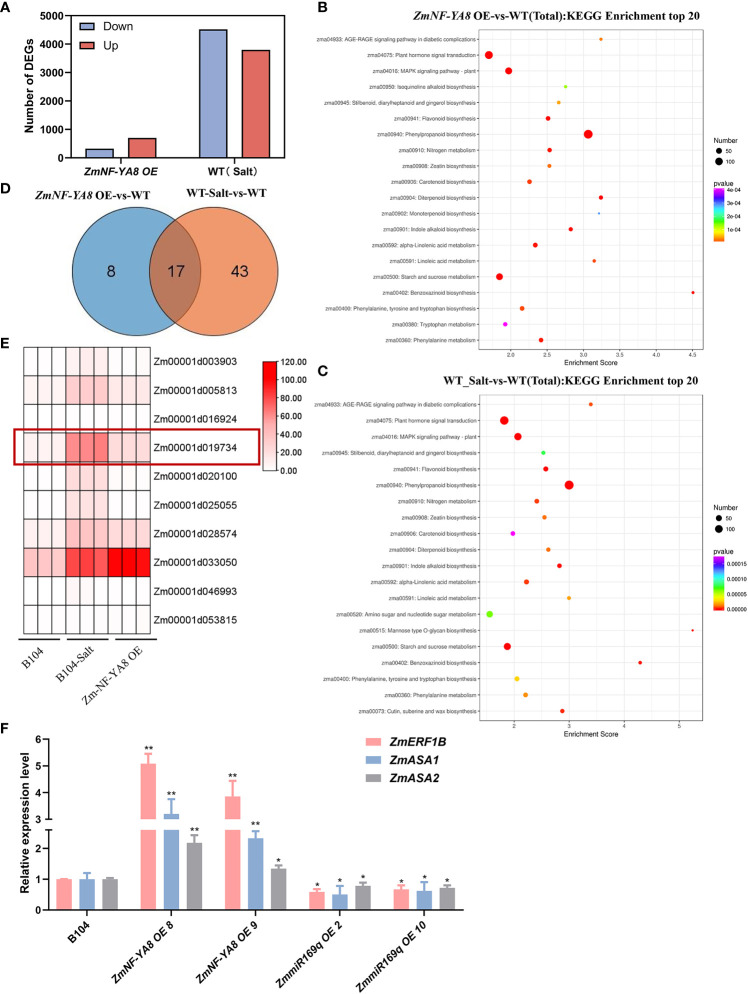
Identification of ZmNF-YA8 target gene through RNA-seq **(A)** The number of differentially expressed genes (DEGs) between wild type (B104), B104-Salt and overexpression (ZmNF-YA8-OE) lines were identified using a significant cutoff of P < 0.01, and a fold change (FC) > 2. **(B, C)** Kyoto Encyclopedia of Genes and Genomes (KEGG) enrichment analysis showed that the significant enrichment pathways in ZmNF-YA8-OE-*vs*-WT and WT_Salt-*vs*-WT. **(D)** Wayne diagram shows that there are 17 DEGS overlapping in the planthomone signature transmission pathway of ZmNF-YA8-OE *vs* B104 and B104 Salt *vs* B104. **(E)** Differential expression of genes contain CCAAT box on promoter were identified. **(F)** Relative expression levels of *Zm-ERF1B, Zm-ASA1* and *Zm-ASA2* in B104, ZmmiR169q-OE and ZmNF-YA8-OE transgenic line under 125 mm NaCl treatment conditions. Actin1 was used as the reference gene for quantitative PCR. Values are expressed as means ± SD of three biological repeats. Asterisks indicate significant differences (*, P < 0.05 **; P < 0.01; unpaired Student’s two-tailed t-test).

RNA-seq analysis was performed on young roots from NF-YA8-OE, B104, and 104-S (with salt treatment) to investigate the underlying molecular mechanism of salt tolerance conferred by the ZmmiR169q/*ZmNF-YA8* module. We identified a total of 1,023 DEGs between ZmNF-YA8-OE and B104, with 702 upregulated, and 322 downregulated. Between B104-S and B104, 8,316 DEGs were detected, of which 3,799 were upregulated and 4,517 were downregulated (**Figure 5A**). Kyoto Encyclopedia of Genes and Genomes (KEGG) enrichment analysis of the DEGs revealed that significant enrichment pathways were mainly related to plant hormone signal transduction (**Figures 5B, C**). Further analysis of the genes involved in the plant hormone signal transmission pathways revealed that, compared to B104, ZmNF-YA8-OE had 25 upregulated DEGs and B104-S had 60 upregulated DEGs. Among them, 17 were shared by these two groups (**Figure 5D**), indicating that these genes may be involved in the regulatory pathway of ZmNF-YA8 under salt stress conditions. Since ZmNF-YA8 acts by binding to the CCAAT-box in the promoter of its target gene, we investigated the promoters of these 17 genes and discovered the CCAAT-box in the promoters of ten genes (**Figure 5E**). Four out of these ten genes exhibited significant upregulation in ZmNF-YA8-OE plants, including Zm00001d005813, Zm00001d033050, Zm00001d028574, and Zm00001d019734, which encode TIFY, a Jasmonate ZIM domain-containing protein, serine/threonine phosphatase, and an auxin synthesis-related transcription factor gene ZmERF1B, respectively. The Arabidopsis homolog of the latter gene was found to inhibit ethylene-mediated primary root growth by regulating auxin biosynthesis (Mao et al., 2016). qRT-PCR verification demonstrated that the expression of *ZmERF1B* was significantly higher in ZmNF-YA8-OE8 and ZmNF-YA8-OE9 than in B104 in response to salt treatment. In contrast, *ZmERF1B* expression in miR169q-OE2 and miR169q-OE10 transgenic lines was significantly lower than that in B104 (**Figure 5F**), well in line with RNA-seq analysis.

The expression of *ZmASA1* and *ZmASA2* was significantly higher in the ZmNF-YA8-OE line than in B104, but significantly lower than in B104 in the miR169q-OE line (**Figure 5F**).

### 3.6 ZmNF-YA8 activates ZmERF1B transcription by binding to CCAAT box of its promoter

**Figure 6:**
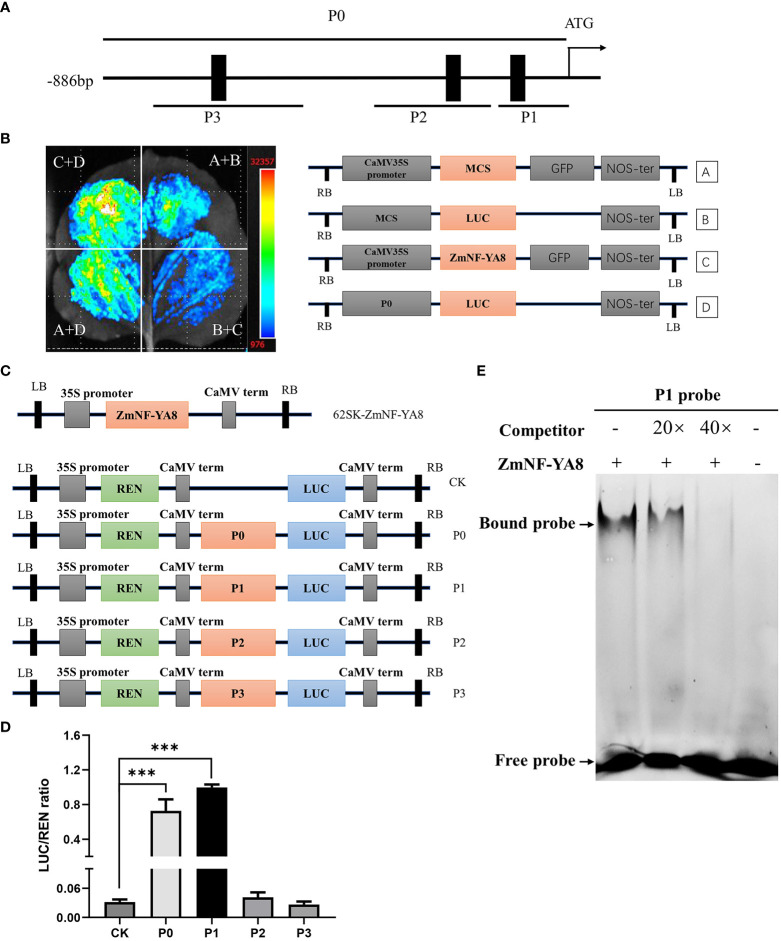
ZmNF-YA8 can activate *ZmERF1B* transcription through directly binding to its promoter sequence **(A)** Schematic diagrams of putative ZmNF-YA8 binding site (CCAAT) in the promoter of *ZmERF1B*. Black boxes indicate the positions of the CCAAT box. P0-P3 are fragments of the ZmERF1B promoter. **(B)** The activation of ZmNF-YA8 activated expression of report gene that contained the promoter of *ZmERF1B* in *Nicotiana benthamiana* leaves. A and B are empty vectors, C is 35S::ZmNF-YA8 effector vectors, D is ZmERF1B promoter::LUC reporter vector. Three biological replicates were performed with similar results. **(C)** Schematic diagram showing the effectors and reporter used in the transient transcriptional activity assays in maize protoplasts. REN, Renilla luciferase; LUC, firefly luciferase. **(D)** The LUC : REN ratio represents the relative activity of the promoter fragments including each CCAAT box of *ZmERF1B*. The expression level of REN was used as an internal control. Data are values of three independent experiments. Error bars indicate ± SD (n ≥ 3). ***, P < 0.001; one-way ANOVA followed by Scheffe’s multiple comparison tests. **(E)** EMSA assay for binding to CCAAT box sequence in the promoter of *ZmERF1B* by ZmNF-YA8 protein *in vitro*. Competition for the 5’FAM labeled promoter region was performed by adding an excess of unlabeled probe (Competitor). Three biological replicates were performed with similar results.

A question arises as to whether ZmNF-YA8 acts as a direct regulator of ZmERF1B. Three putative CCAAT-box motifs were located in the putative promoter region (approximately 1.5 kb upstream of TIS) of ZmERF1B, at -68 bp, -134 bp, and -650 bp upstream of TIS (**Figure 6A**). A series of promoter deletions were synthesized, including P0 that contains all three CCAAT boxes, P1 that contains the first proximal CCAAT boxes, P2 that contains the second proximal CCAAT box, and P3 that contains the distal CCAAT box. The P0 promoter was cloned preceding the LUC gene as its regulatory sequence, forming ZmERF1B promoter:LUC effector vectors (**Figures 6B-D**). *N. benthamiana* leaves transiently expressing the LUC driven by the P0 promoter showed that ZmNF-YA8 could directly bind to the promoter of *ZmERF1B* and enhance its expression (**Figure 6B**).

The pGreen62-SK-ZmNF-YA8 gene cassette was turned on when it was co-expressed with P0 and P1 in maize protoplasts (**Figure 6D**). This was shown by the LUC assay using a dual LUC reporter in maize protoplasts. By contrast, P2 and P3 did not activate LUC expression (**Figure 6D**). It is therefore conceivable that ZmNF-YA8 can bind directly to the P1 CCAAT box in the ZmERF1B promoter and boost its expression.

We performed EMSA experiments to better understand the interaction between ZmNF-YA8 and the P1 CCAAT box. It was shown that the ZmNF-YA8 protein could specifically bind to the P1 CAATT box, as evidenced by the presence of a lagging band during electrophoresis that was reduced in intensity by the addition of an unlabeled competitive probe (20 X) and eliminated entirely at 40 X (**Figure 6E**). As shown above, ZmNF-YA8 was able to directly bind to P1 of the ZmERF1B promoter *in vitro* and *in vivo*, resulting in the activation of the expression of this gene.

### 3.7 Discussion

**Figure 7:**
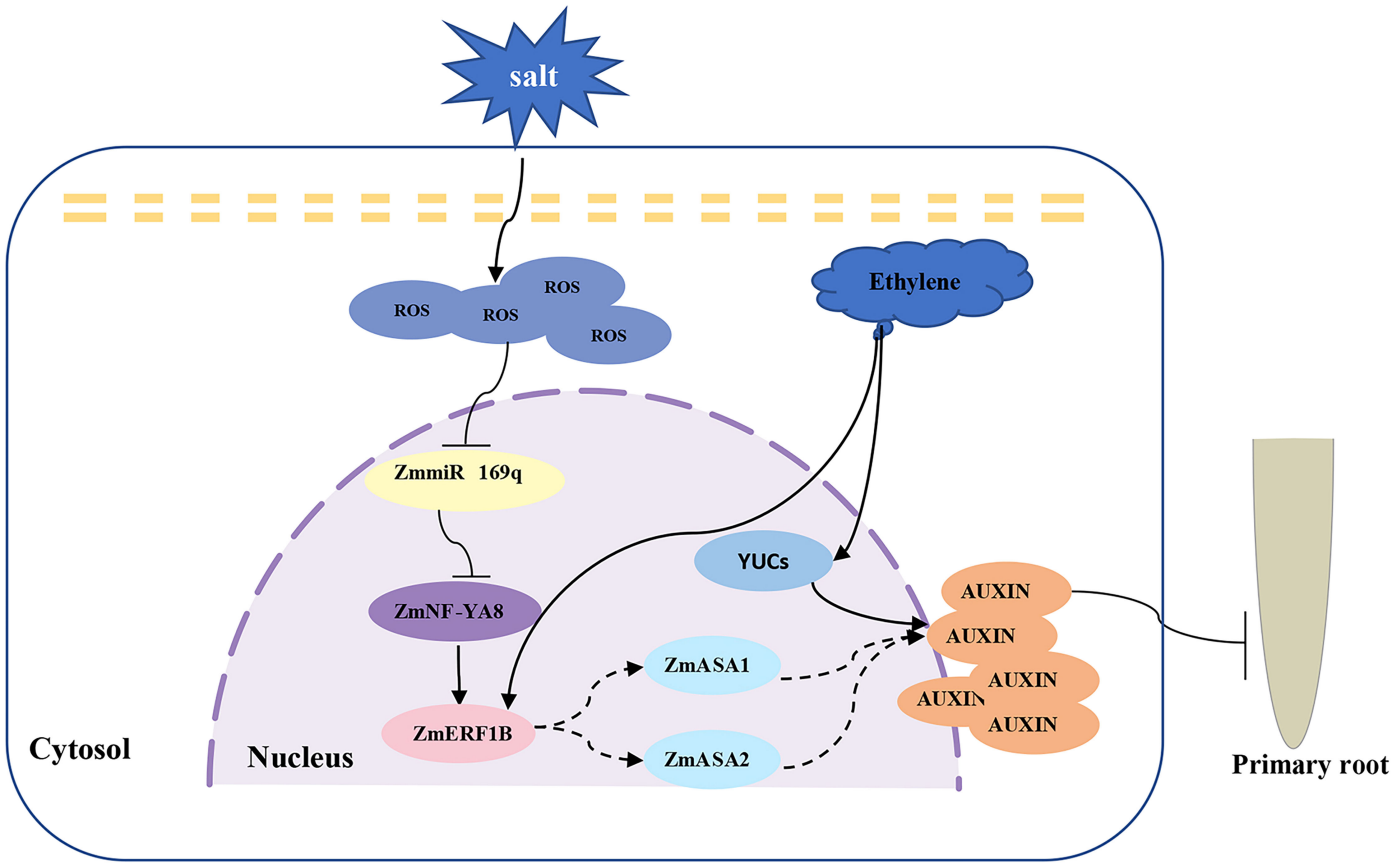
Proposed working model of ZmmiR169q/ZmNF-YA8 module regulating maize taproot growth under salt stress. Salt stress inhibited the expression of ZmmiR169q, led to the up-regulation of its target gene ZmNF-YA8, activated the expression of ZmERF1B, promoted the synthesis and accumulation of auxin in root tips, and inhibited the growth of primary roots.

Salt stress is an abiotic stress that has a significant impact on crop development and ultimately reduces crop productivity. Root structure is significantly affected by salt stress, as exemplified in Arabidopsis, whose primary root development and extension were significantly hindered as a result of size reduction in the root meristem (West et al., 2004b). Studies have also shown that the change in root length is not only dependent on the length of the cells in the root elongation zone but is also related to the number of cells (Liu et al., 2015a). In addition, there is a certain relationship between the growth of the tap root and the lateral root under salt stress. Under low-concentration salt stress (below 25 mmol/L NaCl), the elongation of primary roots was promoted, but lateral root elongation was inhibited. On the contrary, primary root growth was severely inhibited while the elongation of lateral roots was stimulated in response to a high salt concentration. The molecular regulation mechanisms that underpin such an observation are intriguing yet remain elusive (Wang et al., 2009b). In Arabidopsis, salt stress activates the JA signaling pathway through JAR1 and the proteasome in the root meristematic zone, inhibiting root cell elongation (Valenzuela et al., 2016). It was reported that the expression of the auxin-regulated genes (DR5-GUS) in Arabidopsis increased in response to salt treatment, which was corroborated by the increased auxin accumulation in root tips under salt treatment, leading to the supposition that the accumulation of auxin is conducive to root growth inhibition (Jiang et al., 2016). Recent studies have revealed that salt stress significantly dwindles the size of Arabidopsis root meristems by downregulating the expression of the *PIN* gene, thereby reducing auxin levels. Salt stress promotes the accumulation of abscisic acid (ABA) in rice primary roots, thereby activating the *EXPANSIN* gene to inhibit cell proliferation but promote cell expansion in root meristems, and ultimately restrict the elongation and promote the expansion of primary roots (Huang et al., 2021). This is consistent with reports that the total amount of IAA in rice was significantly increased under salt stress, along with a significant rise in IAA synthesis activity (Isobe and Miyagawa, 2022). It is believed that decreased cell elongation in the root elongation zone contributes to the suppression of primary root elongation in maize under salt stress (Bustos et al., 2008). However, how salt stress regulates root meristem development in maize remains unknown. The current study emphatically suggests that auxin is involved in the growth and development of maize primary taproots under salt stress. It has been shown that salt stress increases the expression of *ZmNF-YA8* by inhibiting transcription of its negative regulator, pre-miR169q (Xing et al., 2022). As a result, ZmNF-YA8 binds and activates the transcription of *ZmERF1B*, activating the expression of its downstream target genes *ZmASA1* and *ZmASA2*, which promote the synthesis and accumulation of auxin. The high concentration of auxin inhibits the growth of the maize primary root meristem, leading to shortening of the primary roots and a concomitant enhancement in salt tolerance (**Figure 7**).

It is well established that ethylene promotes auxin synthesis, transport of auxin to the elongation zone, and root tip auxin accumulation (Yau et al., 2004; Muday et al., 2012; Mao et al., 2016). While the connection between ethylene and auxin has been extensively explored in Arabidopsis, the roles of ethylene and auxin in maize root elongation remain unknown. ERF1 plays a key role in ethylene-induced inhibition of Arabidopsis primary root growth and acts as a crossover node in the elongation of primary roots by ethylene and auxin. Ethylene inhibits primary root elongation by promoting auxin biosynthesis in MZ and TZ through ASA1, ASB1, TAA1/TARs, and YUCs. A recent study has shown that ERF109, another member of the ERF family, is highly responsive to JA signaling, directly regulates ASA1 and YUC2, and mediates the crosstalk between JA and auxin biosynthesis (Cai et al., 2014). Our relative gene expression results showed that ZmASA1 and ZmASA2 may be direct targets of ZmERF1B, while ZmASA1 and ZmASA2 further regulate auxin content. In this report, we demonstrate that ZmNF-YA8 activates the transcription of *ZmERF1B*, which is a downstream transcription factor in the ethylene signaling pathway, leading to the synthesis and accumulation of auxin in root tips. Our findings reveal a possible cross-regulatory mode of ethylene and auxin in maize root elongation, in which ZmNF-YA8 plays a vital role in the homeostatic regulation of stress tolerance and growth development in plants under stress conditions.

NF-YA, NF-YB, and NF-YC are members of the NF-Y family of transcription factors, which are ubiquitous heterotrimeric complexes that govern plant growth, development, and stress responses. The number of NF-Y subunits is significantly greater in higher plants than in animals and fungi. The NF-Y complex regulates the expression of target genes through direct binding to the CCAAT box in the gene’s promoter region (Leyva-Gonzalez et al., 2012; Petroni et al., 2012). The NF-Y family plays key roles in the establishment of symbiotic root nodules (Baudin et al., 2015), primary roots (Sorin et al., 2014), photosynthesis, photomorphogenesis, and reproductive development (Sun et al., 2014; Bai et al., 2016). Previous studies have shown that ROS accumulated during salt stress inhibit ZmmiR169q, increasing the expression of the ZmmiR169q target gene *ZmNF-YA8*, which in turn activates the transcription of the high-efficiency antioxidant gene *ZmPER1*, imparting ROS scavenging *in vivo*, and ultimate salt tolerance in maize. In light of these findings, we speculate that ZmmiR169q/ZmNF-YA8 may be in a core regulatory position in plant responses to salt stress and play a vital role in homeostatic control of root growth and development and salt tolerance.

In conclusion, our findings demonstrate that the ZmmiR169q/ZmNF-YA8 module in maize can regulate a series of genes in response to salt stress, resulting in alterations in phytohormone accumulation and a cascade of signal transduction events. However, hormone crosstalk is not linear and should be analyzed within a multidimensional framework, considering the spatio-temporal relationship between the two hormones and their interdependence with other hormones. For instance, auxin and cytokinin primarily regulate the proliferation and differentiation of the meristems. While auxin maintains cell elongation and growth, cytokinin drives cell division processes and sustains the cell proliferation (Zhang and Yuan, 2014). In this study, by measuring the IAA content in maize root tips, we discovered that auxin accumulation in primary root tips resulted in a decreased number of cells in the meristem area, leading to shorter root lengths. The potential relationship between this observation and cytokinin merits further investigation.

The molecular processes governed by the ZmmiR169q/ZmNF-YA8 module underlies the modification of root structure and development patterns, allowing plant roots to access wider regions of the soil environment or evade potentially hazardous locations to survive and thrive under abiotic stress. Our study not only expands our current understanding of the molecular mechanism behind plant salt tolerance achieved by altering root structure, but also offers a valuable perspective for enhancing crop productivity in the face of soil environmental challenges through the homeostatic and synergistic regulation of plant growth, development, and abiotic stress tolerance.

## Data availability statement

The datasets presented in this study can be found in online repositories. The names of the repository/repositories and accession number(s) can be found below: NCBI SRA database, accession number: PRJNA942448.

## Author contributions

LW and LX designed the research and wrote the paper. LX performed observation of taproot length, qRT-PCR, EMSA, *In situ* hybridization, cytological observation, IAA content measurement, transactivation assay, dual-luciferase assay. LZ and ZZ contributed to protein expression, HZ helped perform salt treatment, YzL and YL contributed to association statistical analysis. All authors contributed to the article and approved the submitted version.

## Supplementary material

Supplementary Table 1 List of primers used in this study
